# Neuroprotective properties of anti-apoptotic BCL-2 proteins in 5xFAD mouse model of Alzheimer’s disease

**DOI:** 10.1016/j.ibneur.2023.02.005

**Published:** 2023-02-23

**Authors:** D. Chernyuk, M. Callens, M. Polozova, A. Gordeev, M. Chigriai, A. Rakovskaya, A. Ilina, E. Pchitskaya, C. Van den Haute, T. Vervliet, G. Bultynck, I. Bezprozvanny

**Affiliations:** a Laboratory of Molecular Neurodegeneration, Peter the Great St. Petersburg State Polytechnic University, Saint Petersburg, Russia; b KU Leuven, Laboratory of Molecular & Cellular Signaling, Department of Cellular & Molecular Medicine, Campus Gasthuisberg O/N-I bus 802, Herestraat 49, BE-3000 Leuven, Belgium; c KU Leuven, Research Group for Neurobiology and Gene Therapy, Department of Neurosciences, Campus Gasthuisberg O/N-5 box 1023, Herestraat 49, BE-3000 Leuven, Belgium; d Leuven Viral Vector Core, BE-3000 Leuven, Belgium; e Department of Physiology, UT Southwestern Medical Center at Dallas, Dallas, TX, USA

**Keywords:** Alzheimer's disease, Ca^2+^ signaling, Bcl-2, Synaptic contacts, Amyloid plaques, 5xFAD

## Abstract

Alzheimer's disease (AD) is the most common cause of dementia. An early feature of the AD pathology is the dysregulation of intracellular Ca^2+^ signaling in neurons. In particular, increased Ca^2+^ release from endoplasmic reticulum-located Ca^2+^ channels, including inositol-1,4,5-trisphosphate type 1 receptors (IP_3_R1) and ryanodine receptors type 2 (RyR2), have been extensively reported. Known for its anti-apoptotic properties, Bcl-2 also has the ability to bind to and inhibit the Ca^2+^-flux properties of IP_3_Rs and RyRs. In this study, the hypothesis that the expression of Bcl-2 proteins can normalize dysregulated Ca^2+^ signaling in a mouse model of AD (5xFAD) and thereby prevent or slow the progression of AD was examined. Therefore, stereotactic injections of adeno-associated viral vectors expressing Bcl-2 proteins were performed in the CA1 region of the 5xFAD mouse hippocampus. In order to assess the importance of the association with IP_3_R1, the Bcl-2^K17D^ mutant was also included in these experiments. This K17D mutation has been previously shown to decrease the association of Bcl-2 with IP_3_R1, thereby impairing its ability to inhibit IP_3_R1 while not affecting Bcl-2′s ability to inhibit RyRs. Here, we demonstrate that Bcl-2 protein expression leads to synaptoprotective and amyloid-protective effects in the 5xFAD animal model. Several of these neuroprotective features are also observed by Bcl-2^K17D^ protein expression, suggesting that these effects are not associated with Bcl-2-mediated inhibition of IP_3_R1. Potential mechanisms for this Bcl-2 synaptoprotective action may be related to its ability to inhibit RyR2 activity as Bcl-2 and Bcl-2^K17D^ are equally potent in inhibiting RyR2-mediated Ca^2+^ fluxes. This work indicates that Bcl-2-based strategies hold neuroprotective potential in AD models, though the underlying mechanisms requires further investigation.

## Introduction

Alzheimer's disease (AD) is the most common neurodegenerative disease. In AD, the cells of the hippocampus, the area of the brain responsible for the formation and storage of memory, are primarily affected. Hence, the main manifestation of this disease is a progressive memory loss, eventually leading to dementia and a complete loss of the ability to self-service. At present, a complete understanding of the causes of the onset and development of AD has not been achieved, therefore, modern methods of therapy only slightly alleviate the symptoms, but so far do not allow either to stop or slow down the development of the disease. The main physiological sign of AD is the formation of toxic amyloid plaques in the brain (Hardy and Higgins, 1992, Nussbaum and Ellis, 2003). Despite the ongoing development of anti-amyloid therapy, no clinical trials have been successful to date. Therefore, more and more scientists agree that a more detailed study of the physiological and pathological role of beta-amyloid is needed, as well as the search for new signaling pathways involved in the pathogenesis of AD and new objects of potential therapeutic effect in addition to amyloid β.

In 2009, it was suggested that one of the most important roles in the pathogenesis of neurodegenerative diseases is played by changes in neuronal Ca^2+^ signaling, which are also observed during normal aging (Bezprozvanny, 2009). Neurons are very sensitive to changes in intracellular Ca^2+^ homeostasis, so even the smallest anomalies in Ca^2+^ signaling can lead to devastating consequences over a long period of time. Dysregulation of Ca^2+^ signaling mechanisms is one of the earliest changes at the molecular level in the etiology of AD. Mutations in presenilin proteins (PSEN) associated with the familial form of AD, have been shown to mediate Ca^2+^ release from the endoplasmic reticulum (ER) either directly (Tu et al., 2006, Supnet and Bezprozvanny, 2011) or by modulating the activity of ER-resident Ca^2+^-release channels (Kasri et al., 2006, Wu et al., 2013, Shilling et al., 2014). The main channels responsible for Ca^2+^ release from the ER into the cytoplasm are inositol 1,4,5-trisphosphate receptors (IP_3_Rs) (Prole and Taylor, 2016) as well as ryanodine receptors (RyRs) (Lanner, et al., 2010). The latter are activated by Ca^2+^ itself through Ca^2+^-induced Ca^2+^ release, which enables the amplification of cytosolic Ca^2+^ signals. IP_3_R1 is the predominantly expressed isoform in the brain (Baker et al., 2017, Rossi and Taylor, 2018). IP_3_R1-mediated Ca^2+^ release is involved in many cellular processes including cell survival, apoptosis, autophagy, and many others (Taufiq et al., 2005, Yamazaki et al., 2012, Ivanova et al., 2014, Kania et al., 2017). An important part of these processes is the “quasi-synaptic” transmission of Ca^2+^ signals from the ER to mitochondria through the so-called mitochondria-associated ER membranes (MAMs). This signaling occurs via the IP_3_R on the ER side and the voltage-gated anion channel and mitochondrial Ca^2+^ uniporter on the mitochondrial side (Szymanski et al., 2017). Ca^2+^ transfer to mitochondria is necessary, in particular, to maintain the ATP synthesis and in general for the normal functioning of cells (Bravo et al., 2011, MacVicar et al., 2015). Both an increase and a decrease in IP_3_R-mediated Ca^2+^ transfer into mitochondria leads to their dysfunction and induces cell death (Decuypere et al., 2011).

IP_3_R function is tightly regulated by accessory proteins. It has been extensively documented that the IP_3_R is a target of anti-apoptotic B-cell lymphoma 2 (Bcl-2) proteins, including Bcl-2 and Bcl-XL (Rong et al., 2009, Monaco et al., 2012, Callens et al., 2021, Rosa et al., 2022). Anti-apoptotic Bcl-2 proteins, which are characterized by four Bcl-2-homology (BH) domains, are well known for their action at the level of the mitochondria, where they scaffold and neutralize Bax and Bak proteins and thus prevent mitochondrial outer member permeabilization (Singh et al., 2019, Vandenabeele et al., 2022). As such, anti-apoptotic Bcl-2 prevents cytochrome c release and downstream activation of apoptosis executioners such as caspase 3. However, Bcl-2 proteins also act at the ER Ca^2+^ stores, where they inhibit IP_3_Rs and thus pro-apoptotic Ca^2+^ release events (Ivanova et al., 2020). The N-terminal Bcl-2-homology domain 4 (BH4 domain) of Bcl-2 is necessary and sufficient for Bcl-2-mediated inhibition of IP_3_Rs and Ca^2+^-driven apoptosis (Rong et al., 2009, de Ridder et al., 2021).

It was shown that Bcl-2 binds to and inhibits both IP_3_R and RyR through its BH4 domain (Rong et al., 2009, Monaco et al., 2012, Vervliet et al., 2014). An important role for lysine 17 in the BH4 domain concerning its binding to the IP_3_R was described (Monaco et al., 2012). Hence, mutating this lysine to an aspartic acid (K17D) reduced Bcl-2′s interaction with the IP_3_R and impaired Bcl-2′s ability to inhibit IP_3_Rs but did not affect Bcl-2′s ability to bind to and inhibit RyRs, validated for RyR1 and RyR3 isoforms (Monaco et al., 2012, Vervliet et al., 2014). This Bcl-2^K17D^ mutant, which can still inhibit RyRs but not IP_3_Rs, was included in the present study to investigate whether the observed effects by Bcl-2 overexpression can be linked to Bcl-2′s ability to bind and inhibit IP_3_Rs. In addition, Bcl-2 family proteins are associated with AD (Callens et al., 2021). Overexpression of Bcl-2 in the transgenic AD mice brain slowed down the progression of cognitive impairment, while the formation of extracellular plaques decreased (Rohn et al., 2008). It was suggested that the neuroprotective effect of Bcl-2 was associated with the inhibition of apoptosis executioners thereby reducing the caspase 9 and caspase 3 activity. However, the question of the involvement of Ca^2+^ signaling mechanisms in the observed effect remains open and is relevant. Therefore, Bcl-2 may play a dual role in neuronal cells to counteract the development of AD, firstly as an inhibitor of Ca^2+^ release, and secondly, as an inhibitor of mediated apoptosis.

Thus, presenilin mutations associated with the familial form of AD affect intracellular Ca^2+^ signaling, leading to excessive Ca^2+^ release from the ER. This study is aimed at using Bcl-2-based strategies to counteract AD by exploiting Bcl-2′s ability to suppress the transmission of IP_3_R/RyR-driven Ca^2+^-dependent signals that are dysregulated in the AD.

## Material and methods

### Mice model of Alzheimer’s disease

5xFAD mice (B6SJLF1/J background, Jackson Labs: stock #006554) were used to study AD. 5xFAD is a transgenic mouse line with 5 mutations - three mutations in the human APP protein (Swedish (K670N/M671L), Florida (I716V), London (V717I)) and two mutations in the presenilin 1 protein (M146L, L286V). In mice of this line, neuronal death is observed starting from the age of 6 months, which correlates with the accumulation of beta amyloid and caspase 3 activation in large pyramidal neurons of the brain (Rodriguez et al., 2008). Also, crossing and breeding of two mouse lines, 5xFAD and M-line, was carried out. M-line mice (hy1-GFP-M. GFP - green fluorescent protein) are convenient for the morphological analysis of neuronal cells, since they express GFP-fluorescently labeled neurons, which makes it possible to study cell morphology in detail.

Animal experiments were carried out in accordance with the National Institute of Health Guide for the Care and Use of Laboratory Animals (NIH Publications No. 80–23) revised 1996 or the UK Animals (Scientific Procedures) Act 1986 and associated guidelines, or the European Communities Council Directive of 24 November 1986 (86/609/EEC). Mice are kept in the vivarium of the Laboratory of Molecular Neurodegeneration of Peter the Great St. Petersburg Polytechnic University with a 12-hour light cycle with *ad libitum* access to food and water.

Formal approval to conduct the experiments described has been obtained from the animal subjects review board of Peter the Great St. Petersburg Polytechnic University and could be provided upon request. All efforts were made to minimize the number of animals used and their suffering.

### Injection of viral vectors

For overexpression of Bcl-2 in the CA1 hippocampus area, adeno-associated viral vectors (AAV) under the control of the neuronal synapsin protein promotor were designed: control AAV2/7-mCherry (below Control), AAV2/7–3xFLAG-Bcl-2-P2A-mCherry (below Bcl-2), encoding the native Bcl-2 protein, and AAV2/7–3xFLAG-Bcl-2^K17D^-P2A-mCherry (below Bcl-2^K17D^), encoding the mutant form of the Bcl-2 protein. These viral vectors enable the expression of both Bcl-2 and mCherry as 2 separate proteins. The AAV CMVie synpasin-intron-3xFLAG-mCherry-Bcl-2 was also developed to express mCherry-fused Bcl-2, enabling the analysis of Bcl-2 localization through mCherry visualization.

Bilateral stereotaxic surgery was used to deliver AAV overexpressing the Bcl-2 proteins into the hippocampus of adult mice at the coordinates for injection into the CA1 hippocampus area: AP = −2.1, mL = + 2.0 DV = −1.9. The concentrations and expression level of each of the viral vectors were tested and following injection dosed were selected:1 µl for AAV2/7–3xFLAG-Bcl-2-P2A-mCherry 6.63E+ 11 GC/mL, 0.6 µl for AAV2/7–3xFLAG-Bcl-2^K17D^-P2A-mCherry 1.20E+ 12 GC/mL, 0.5 µl for AAV2/7-mCherry 3.15E+ 12 GC/mL and 2 µl for CMVie synapsin-intron-3xFLAG-mCherry-Bcl-2 3.07E+ 11 GC/mL. After 30–60 days, when the target proteins were produced in sufficient quantities, further experiments were carried out.

### Co-immunoprecipitation

The interaction of 3xFLAG-Bcl-2 and 3xFLAG-Bcl-2^K17D^ proteins with IP_3_R1 and RyR2 was determined by immunoprecipitation. For immunoprecipitation, 30 days after bilateral injection, perfusion with phosphate-buffered saline (PBS) solution occurred, the hippocampus was crushed and transferred to a homogenizer for cell lysis in a lysis buffer containing 25 mM tris(hydroxymethyl)aminomethane (Tris), 150 mM sodium chloride, 10 mM ethylenediaminetetraacetic acid, 1 % NP40 and 1 mg/mL protease inhibitor solution (Roche), pH 7.6 at + 4 °C. The cells were lysed for 5 h and then centrifuged at 20,000 g at + 4 °C for 20 min, one sample always included 2 hippocampi from one mouse, which is about 0.3 g. At this time, pre-washed with a cold PBS solution, G-protein-conjugated magnetic particles are incubated for 2.5 h at a temperature of + 4 °C with monoclonal antibodies to Flag tag (1:100 dilution, Sigma Aldrich, Cat. No. F1804) or with control antibodies. Immunoglobulins of an animal-producer similar to immunoglobulins antibodies were used as control antibodies to detect nonspecific protein binding. After centrifugation, the supernatant containing the target proteins is transferred to the antibodies deposited on magnetic particles and then incubated for 16 h at + 4 °C with constant rotation of the samples on a rotator. At the end of the incubation time, the magnetic particles with antibodies and the protein complex were separated from the rest of the cell lysate using a miniature magnetic stand. Next, a series of washes with a lysis buffer was performed to remove non-specifically bound proteins. After that, the protein complex with magnetic particles was eluted for further analysis by incubation in 1x Laemmli's buffer at + 90 °C for 5 min. The sample containing the target proteins in Laemmli's buffer was separated from the magnetic particles using a miniature magnetic rack and further analyzed using Western Blot analysis. Cell lysates were separated on a 3–8% polyacrylamide gradient gel under denaturing conditions, transferred to a membrane, and incubated with primary antibodies to IP_3_R1 (1:1000 dilution, Abcam, Cat. No. ab264281), to RyR2 (1:500 dilution, DSHB, Cat. No. 34 C-c) and to Bcl-2 (1:200 dilution, Invitrogen, Cat. No. MA5–11757) for 16 h. The membrane was washed several times with a buffer containing 20 mM Tris, 150 mM sodium chloride and 0.1 % polysorbate 20, pH 7.6 and incubated with secondary antibodies conjugated with horseradish peroxidase (1:200 dilution, cat. No. P0448 "DAKO", USA) for1 hour. Next, the membrane was washed several times and incubated in a solution that induces a chemiluminescence reaction. Finally, the signal was recorded using X-ray film. The expression level of Bcl-2 was used as a control for protein loading in the sample.

### Cell culture

HEK T-Rex RyR2 cells were kindly gifted by Dr. Wayne Chen (Department of Physiology and Pharmacology, University of Calgary, Canada) and were originally described by Liu et al. (2013). Cells were cultured at 37 °C in the presence of 5 % CO_2_ in Dulbecco's Modified Eagle Medium (DMEM) (Gibco) supplemented with 10 % fetal calf serum (Sigma-Aldrich), 1% non-essential amino acids (Gibco), 4 mM L-glutamine, (Gibco) 100 units/mL penicillin (Gibco) and 100 µg/mL streptomycin (Gibco). Cells were cultured in mycoplasma-free conditions and were monthly checked for mycoplasma infection.

### Single-cell Ca^2+^ imaging

HEK T-Rex RyR2 cells were plated in four-chamber 35-mm dishes at a density of 90.000 cells per chamber. 24 h after seeding, cells were transiently transfected with 125 ng DNA/chamber of one of the following plasmids: pCMV24-P2A-mCherry, pCMV24–3xFLAG-Bcl-2-P2A-mCherry or pCMV24–3xFLAG-Bcl-2^K17D^-P2A-mCherry. TransIT-X2® Dynamic Delivery System (Mirus) transfection reagent was added according to the manufacturer’s instructions. Approximately 3 h after transfection, cell medium was supplemented with tetracycline (15 ng/mL) (Sigma-Aldrich) to induce the expression of RyR2. 48 h after transfection, the cells were loaded with Fura-2-AM (AnaSpec) in modified Krebs buffer (135 mM NaCl, 6.2 mM KCl, 1.2 mM MgCl_2_, 12 mM HEPES, pH 7.3, 11.5 mM glucose and 2 mM CaCl_2_) for 30 min at room temperature. Afterwards, cells were allowed to de-esterify at room temperature for another 30 min. Ca^2+^ imaging was performed using Nikon Eclipse Ti2 inverted microscope with a 20x air objective (Nikon, Amstelveen, The Netherlands). 3 mM EGTA (Sigma-Aldrich) was used to chelate extracellular Ca^2+^ and 0.5 mM Caffeine (Sigma-Aldrich) was used to elicit RyR-mediated Ca^2+^ response. The baseline was calculated as the average fluorescence between EGTA and agonist addition. The area under the curve and the peak amplitude (max amplitude-baseline) after agonist addition were calculated.

### Spine morphology analysis

To assess the morphology of dendritic spines in vivo, a line of 5xFAD-M transgenic mice was used, obtained by crossing 5xFAD mice with transgenic M-line mice. 60 days after AAV injection, perfusion was performed with a 4% solution of paraformaldehyde diluted in PBS. Fixed 30 µm thick slices of the brain were placed under coverslips using the mounting medium Aqua Poly/Mount (Polysciences, Inc., USA), after which the morphological analysis of the secondary dendrites of hippocampal CA1 neurons was performed using confocal microscopy. A quantitative analysis of the distribution of dendritic spines by morphological groups was carried out using the freely available Neurostudio software, according to the parameters previously described in (Rodriguez et al., 2008).

### Amyloid β plaques analysis

To quantify amyloid plaques, transcranial perfusion with 4% paraformaldehyde diluted in PBS was performed 60 days after injection. For further immunohistochemical staining, fixed 100 µm thick brain slices were used. During the selection of conditions, the standard protocol for immunohistochemical staining was improved using the HCl antigen retrieval method, as preliminary data showed that the best visualization of amyloid accumulations in brain tissue occurs when pre-enhanced staining by antigen demasking. After 20 min of incubation with 2 N HCl, slices were blocked with 5% BSA diluted in PBS with 0.25 % Triton X-100. Slices were then incubated overnight in a 6E10 primary monoclonal antibody solution (1:1000 dilution, Biolegend Cat# 803001), solution in PBS with 5 % BSA and 0.125 % Triton X-100. After, slices were incubated for two hours with secondary antibodies Alexa Fluor-488 (anti-mouse, Invitrogen, cat. no. A11001) diluted 1:1000 in PBS with 5% BSA and 0.125 % Triton X-100. After that, they were placed under coverslips using Aqua Poly/Mount mounting medium (Polysciences, Inc., USA) and the amyloid plaques were analyzed using a confocal microscope. The percentage of the surface occupied by plaques from the total scanned area was used as parameter to assess the accumulation of β amyloid in the brain using the freely available Icy software.

### Protein colocalization analysis

In order to assess the localization of Bcl-2 and IP_3_R proteins in vivo, the immunohistochemical staining method described above and the expansion microscopy technique were used. A group of wild-type and transgenic 5xFAD mice were injected with the CMVie synpasin-intron-3flag-mCherry-Bcl-2 viral vector at the age of 3.5 months and at the age of 5.5 months perfused with 4% paraformaldehyde in PBS was performed. Next, slices with a thickness of 40–50 µm were made. To enhance the staining, the HCl-antigen retrieval method was used, which is necessary for image analysis using expansion microscopy, since sample expansion leads to a decrease in the concentration of fluorophores and, as a result, the brightness of the resulting signal. Incubation with primary monoclonal antibodies to mCherry proteins (1:200 dilution, Invitrogen, cat. no. MA5–32977) and polyclonal IP_3_R1 (1:300 dilution, Abcam, cat. no. ab5804) was carried out for 16 h, after which there was staining with secondary antibodies Alexa-Fluor 594 (diluted 2 drops per mL, anti-mouse, Invitrogen, Cat. No. R37121) and 488 (diluted 2 drops per mL, anti-rabbit, Invitrogen, Cat. No. R37116) for 2 h. Then, the section was expanded in accordance with the protocol of expansion microscopy to enlarge the sample and visualized using confocal microscopy (Wassie et al., 2019, Derevtsova et al., 2021). Next, the microimages were processed using ImageJ. JACoP (plugin) was used for non-expanded images to identify the matching pixels.

### Statistical analysis

Statistical processing of the obtained results was carried out using the Microsoft Excel and GraphPad Prism software, using Student's t-test, Mann-Whitney U-test, one-way analysis of variance and the Kruskal-Wallis test.

## Results

### Interaction of Bcl-2 and Bcl-2^K17D^ proteins with IP_3_R1 and RyR2

**Fig. 1:**
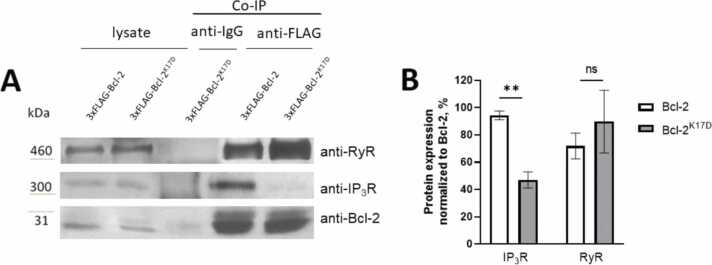
Bcl-2 binds to both IP_3_R and RyR, while Bcl-2^K17D^ interacts only with RyR**.** Results of co-immunoprecipitation of IP_3_R1 and RyR2 from adult mouse hippocampal lysate with antibodies to Bcl-2 protein. A) Western blot analysis of anti-FLAG-based immunoprecipitation assays performed on lysates from 5xFAD mouse hippocampal injected with AAV-3xFLAG-Bcl-2-P2A-mCherry and its mutant form 3xFLAG-Bcl-2^K17D^-P2A-mCherry. The immunoprecipitated samples were analyzed via immunoblotting using antibodies against IP_3_R1, RyR2 and Bcl-2. The numbers on the left indicate the approximate molecular weight. B) Histogram of the relative presence of IP_3_R1 and RyR2 proteins in the immunoprecipitated samples, normalized by the abundance of 3xFLAG-Bcl-2 or 3xFLAG-Bcl-2^K17D^ in that sample. Experiment repeated 3 times. Results are presented as mean ±SEM. ns - no statistically significant difference, t-test ** p < 0.005.

Lysates from mouse hippocampi transduced with AAVs encoding either 3xFLAG-Bcl-2 or 3xFLAG-Bcl-2^K17D^ were used to assess their ability to bind to IP_3_R1 and RyR2 proteins in a neuronal context (Fig. 1). Fig. 1A shows a typical experiment, while Fig. 1B shows the quantitative analysis of independent experiments. The 3xFLAG-Bcl-2 and 3xFLAG-Bcl-2^K17D^ were immunoprecipitated using antibodies directed against FLAG and the presence of IP_3_R1 and RyR2 in the immunoprecipitated sample was determined via immunoblotting. In each experiment 1/15 of the cell lysate (that is about 17–20 µl) was used as input load. Fig. 1A shows a 3xFLAG-Bcl-2^K17D^ overexpression sample as a control co-IP with anti-IgG, in other experiments, a 3xFLAG-Bcl-2 overexpression sample was also tested for anti-IgG, the data is the same as in Fig. 1B, and not shown. An immunoreactive band of about ∼300 kDa (corresponding to the molecular weight of IP_3_R1) in the 3xFLAG-Bcl-2-immunoprecipitated sample was detected in the immunoblot analysis using anti-IP_3_R1, indicating an interaction between wild-type 3xFLAG-Bcl-2 and endogenous IP_3_R1 in the hippocampus. However, the density of the immunoreactive band corresponding to IP_3_R1 was reduced in the 3xFLAG-Bcl-2^K17D^-immunoprecipitated sample (Fig. 1). Hence, consistently with our previous observations (Monaco et al., 2012), the interaction of Bcl-2 with IP_3_R1 depends on the presence of Lys17 in Bcl-2. Using the same approaches but now immunoblotting with anti-RyR antibodies, we demonstrated that both 3xFLAG-Bcl-2 and 3xFLAG-Bcl-2^K17D^ were equally able to interact with endogenous RyR channels (presumably RyR2 as being the most abundant RyR isoform in hippocampus) from the hippocampus (detected as an immunoreactive band of about ∼500 kDa). This indicates that similarly to what has been observed for RyR1 and RyR3 that also the binding of Bcl-2 to RyR2 is not dependent on the presence of Lys17 in Bcl-2.

In summary, we confirmed that in primary hippocampal samples of the 5xFAD mouse, Bcl-2 and the Bcl-2^K17D^ mutant were equally potent to interact with endogenous, neuronal RyR2 channels whereas the binding of endogenous, neuronal IP_3_R1 to Bcl-2^K17D^ is severely impaired compared to its binding to wild-type Bcl-2. These findings correlate with our previous observations (Monaco et al., 2012, Vervliet et al., 2014).

### Caffeine-induced RyR2-mediated Ca^2+^ response in HEK-RyR2 cells overexpressing Bcl-2 or Bcl-2^K17D^

**Fig. 2:**
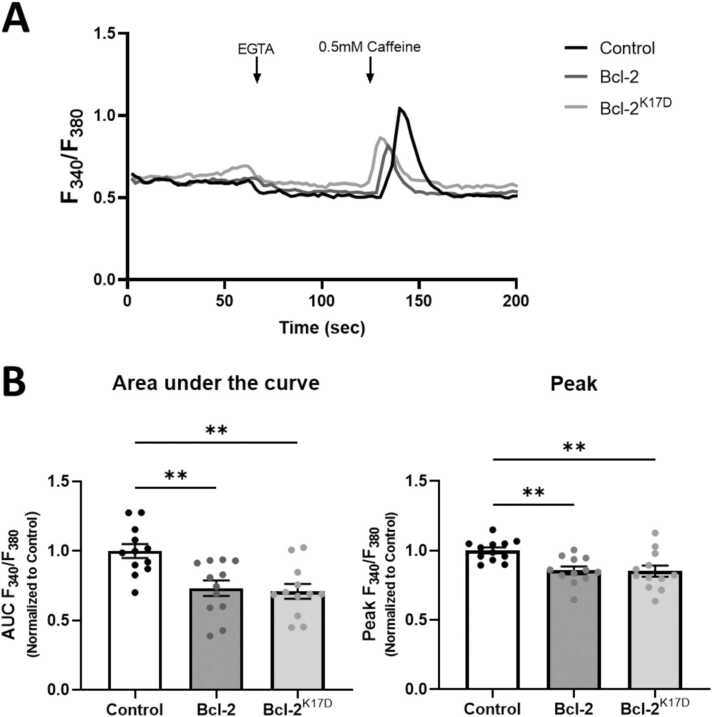
3xFLAG-Bcl-2 and 3xFLAG-Bcl-2^K17D^ are equally potent in inhibiting RyR2-mediated Ca^2+^ responses in HEK T-Rex RyR2 cells**.** HEK T-Rex RyR2 cells were transfected with either pCMV24-P2A-mCherry (control), pCMV24–3xFLAG-Bcl-2-P2A-mCherry (Bcl-2) or pCMV24–3xFLAG-Bcl-2^K17D^-P2A-mCherry (Bcl-2^K17D^). The cells were loaded with Fura2-AM and 0.5 mM Caffeine was used to evoke RyR2-mediated Ca^2+^ response. 3 mM EGTA was used to chelate the extracellular Ca^2+^. A) Average cytosolic Ca^2+^ traces (plotted as F340/F380) recorded from control, 3xFLAG-Bcl-2 or 3xFLAG-Bcl-2^K17D^-expressing HEK Trex RyR2 cells present in one chamber, whereby extracellular Ca^2+^ was first chelated using EGTA and subsequently RyR2-mediated Ca^2+^ release was evoked by 0.5 mM Caffeine. B) The area under the curve and the peak amplitude after agonist stimulation is presented. For each condition, 12 different chambers from 4 independent transfections were measured. Data are represented as averages of chambers; each data point represents one chamber (n = 12). Data is normalized to the average value of the control for each experimental day/transfection. Results are presented as mean ± SEM. Statistically significant differences were determined using one-way ANOVA (**P < 0.005).

While we previously studied the functional impact of Bcl-2 and Bcl-2^K17D^ on RyR1 and RyR3 channels, we never performed such analyses for RyR2 channels. Therefore, we performed live calcium imaging in Fura2-loaded HEK293 T-Rex cells expressing RyR2 channels under the control of a tetracycline-inducible promotor (HEK T-Rex RyR2 cells) and determined the effect of overexpression of Bcl-2 (pCMV24–3xFLAG-Bcl-2-P2A-mCherry) and Bcl-2^K17D^ (pCMV24–3xFLAG-Bcl-2^K17D^-P2A-mCherry) on RyR2-mediated Ca^2+^ release in comparison to overexpressing of the control vector (pCMV24-P2A-mCherry). In these Ca^2+^ recordings, first EGTA was used to chelate extracellular Ca^2+^, thus avoiding Ca^2+^ entry and then caffeine (0.5 mM) was used to evoke RyR2-mediated Ca^2+^ release. The caffeine-evoked Ca^2+^signal in mCherry-positive cells (ensuring the presence of the vector) was quantified by calculating the area under the curve and the peak amplitude. The area under the curve and the amplitude of the caffeine-evoked, RyR2-mediated Ca^2+^signal was reduced in cells expressing either 3xFLAG-Bcl-2 or 3xFLAG-Bcl-2^K17D^. The decrease in caffeine-evoked Ca^2+^ rise was similar between 3xFLAG-Bcl-2 and 3xFLAG-Bcl-2^K17D^, indicating that Bcl-2 and Bcl-2^K17D^ are equally potent in inhibiting RyR2 channels. This is consistent with our previous observations obtained for RyR1 and RyR3 channels (Vervliet et al., 2014) and with our current co-IP results obtained in mouse hippocampi (see Fig. 1). Besides the effect on RyR channels, our previous studies reported the effect of Bcl-2 and Bcl-2^K17D^ on the IP3R, indicating that Bcl-2^K17D^ is impaired in inhibiting IP_3_R-mediated Ca^2+^ release (Monaco et al., 2012, Rosa et al., 2021). Fig. 2.

### Co-localization of Bcl-2 and IP_3_R1 proteins

**Fig. 3:**
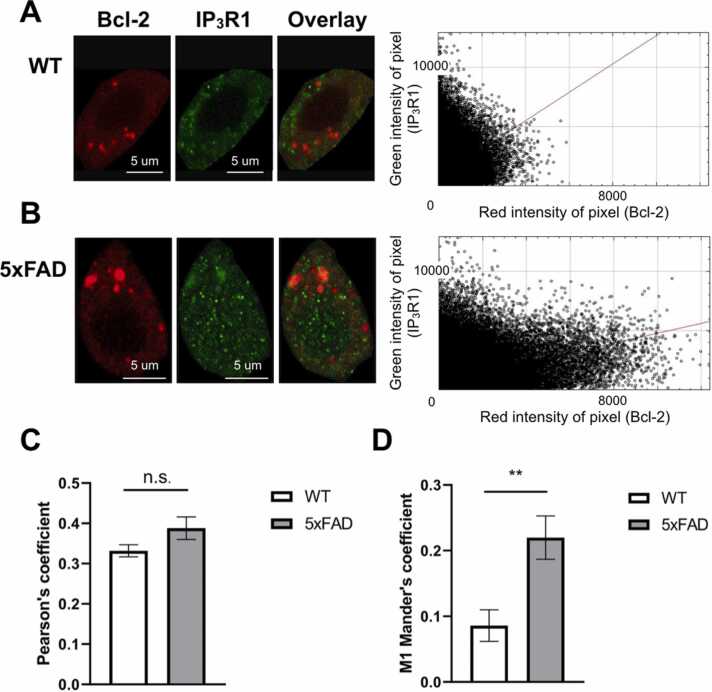
Bcl-2 and IP_3_R1 are located in the soma of the hippocampal neurons of WT and 5xFAD mice in the form of clusters. A) WT and B) 5xFAD mouse hippocampal neuron transduced with mCherry-Bcl-2 (red) and IHC stained with anti-IP_3_R1 antibodies (green), scale bar 5 µm. On the right — cytofluorogram of co-localization between Bcl-2 and IP_3_R (the intensity of a given pixel in the green image is used as the y-coordinate of the scatter plot and the intensity of the corresponding pixel in the red image as the x-coordinate) for each group. C) Pearson’s coefficient, showing variability in one channel caused by variability in the other channel, 5xFAD n = 12, WT n = 11. n.s. — no statistical difference, using Mann-Whitney test. D) Mander’s coefficient, showing the fraction of Bcl-2 overlapping IP_3_R1, 5xFAD n = 12, WT n = 11, ** p < 0,01, using Mann-Whitney test.

**Fig. 4:**
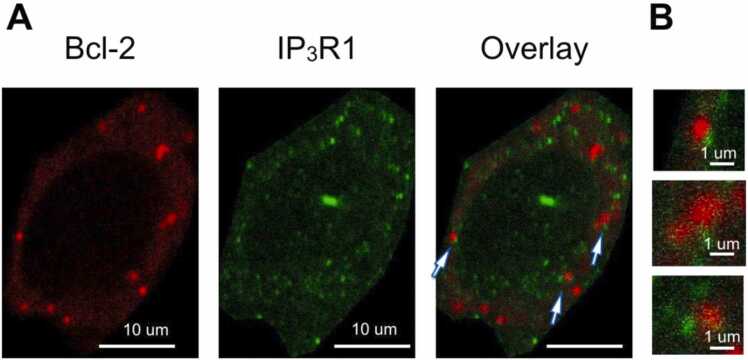
Bcl-2 clusters poorly co-colocalize with IP_3_R clusters in mice hippocampus in vivo, which is confirmed using expansion microscopy. A) Neurons of the hippocampal CA1 region of wildtype mice after immunohistochemical staining and after expansion of samples with polyacrylamide gel, localization of IP_3_R1 and mCherry-Bcl-2-WT, pointers show the clusters that are in contact, scale bar 10 µm. B) Bcl-2 clusters and IP_3_R1 clusters in contact, scale bar 1 µm.

To determine the co-localization of Bcl-2 and IP_3_R1 proteins, 40–50 µm thick slices made from WT and 5xFAD mice injected with CMVie synapsin-intron-3xFLAG-mCherry-Bcl-2 viral vector were stained with antibodies against mCherry and IP_3_R1 proteins and subsequently imaged using confocal and expansion microscopy (Fig. 3, Fig. 4). It was found that both Bcl-2 and IP_3_R1 are located in the soma of the neurons predominantly in the form of clusters (Fig. 3). We studied these proteins co-localization using Manders and Pearson coefficients of the pixel-intensity correlation. Pearson coefficient is 0.332 ± 0.015, n = 11 for wild type and 0.388 ± 0.028, n = 12 for 5xFAD with no significant differences between groups. Manders coefficient is 0.086 ± 0.024, n = 11 for wild type and 0.220 ± 0.033, n = 12 for 5xFAD with difference between groups p < 0,01. Overall, obtained data showed that these proteins poorly co-localize in hippocampal neurons. Using expansion microscopy, which enables to get better resolution, (Fig. 4) we observed some Bcl-2 and IP_3_R1 clusters lying close to each other, forming contacts (Fig. 4).

### Analysis of the morphology of dendritic spines in the hippocampus of 5xFAD mice

**Fig. 5:**
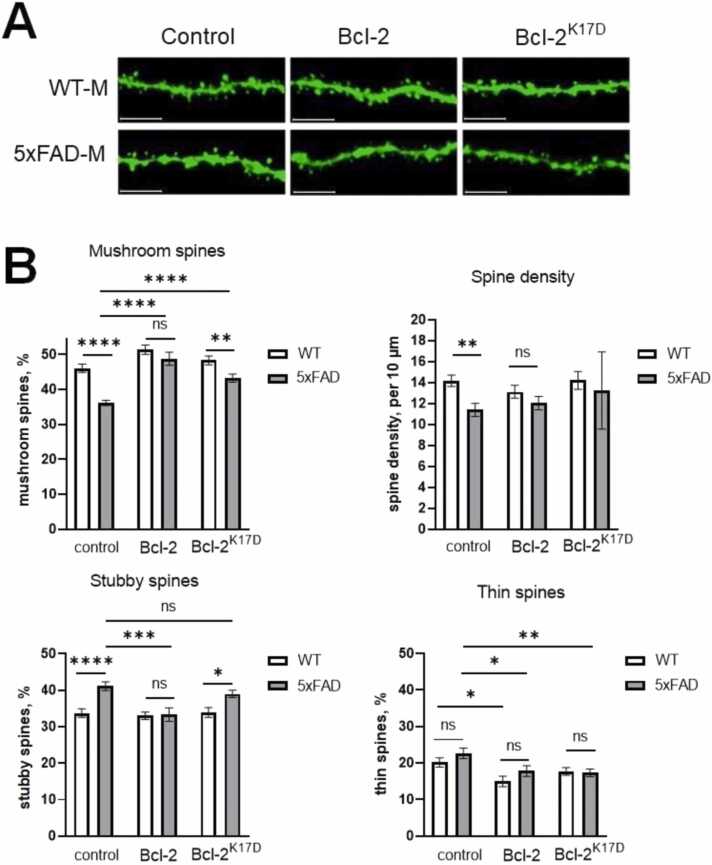
Bcl-2 proteins have synaptoprotective properties**.** Hippocampal slices of wild-type (WT-M) and 5xFAD-M mice injected with Control, 3xFLAG-Bcl-2-P2A-mCherry, 3xFLAG-Bcl-2^K17D^-P2A-mCherry viral vector (in each group n = 21). A) Representative images of dendritic spines in experimental groups. Scale bar 5 µm. B) Histograms illustrating the density of spines and the percentages of mushroom (MS), stubby (SS) and thin spines (TS) in the experimental groups. Results are presented as mean±SEM of the mean, Kruskal-Wallis test **** p < 0.0001,*** p < 0.0005, * * p < 0.005, * p < 0.05.

Analysis of the morphology of dendritic spines was performed on 6-month-old 5xFAD-M and WT-M mice. The results are shown in Fig. 5. According to the data obtained, there is a statistically significant difference in the percentage of mushroom and stubby spines, as well as in the density of dendritic spines between the control mice M-line and 5xFAD-M injected with AAV2/7-mCherry viral vector. Thus, in M-line mice, the percentage of mushroom spines was 46.1 ± 1.2 %, the percentage of stubby spines was 33.7 ± 1.2 %, and the density was 14.2 ± 0.6 spines per 10 µm of dendrite length, while in 5xFAD-M mice these parameters were 36.1 ± 0.8 %, 41.2 ± 1.2 %, and 11.4 ± 0.7 spines per 10 µm of dendrite length, respectively (Fig. 5B). This result is consistent with the literature data (Dorostkar et al., 2015) and has been repeatedly confirmed by the studies of our laboratory (Khavinson et al., 2021).

After injection of the AAV expressing 3xFLAG-Bcl-2-P2A-mCherry into WT-M mice, no changes in dendritic morphology were observed, except for a decrease in the percentage of thin spines, which became 15.0 ± 1.5 %. In 5xFAD-M mice, however, significant changes were observed in the form of an increase in the percentage of mushroom spines to 48.8 ± 1.9 %, a decrease in the percentage of stubby spines to 33.3 ± 1.8 %, and a decrease in the percentage of thin spines to 17.8 ± 1.5 %. At the same time, in groups of wild-type Bcl-2, there is no difference in any of the parameters between WT-M and 5xFAD-M mice. After injection of Bcl-2^K17D^ viral vector into WT-M mice, no significant changes in the morphology of dendritic spines were observed. In 5xFAD-M mice, after injection of the Bcl-2^K17D^ virus, an increase in the number of mushroom- spines to 43.3 ± 1.2 % and a significant decrease in the number of thin spines to 17.3 ± 1.1 % were observed. There were no changes in the percentage of stubby spines and spine density in this group, however, there was a strong scatter in the data on the density of spines (SEM = 3.8 spines per 10 µm dendrite length). In addition, in the Bcl-2^K17D^ injected groups, as well as in the control viral vector injected groups, the difference between WT-M and 5xFAD-M mice in the percentage of mushroom and stubby spines remains. Hence, the overexpression of Bcl-2-WT in the CA1 hippocampus area at the early stages of development of 5xFAD transgenic mice helps to prevent a synaptotoxic effect on hippocampal neurons at a more mature age. There is a significant increase in the percentage of mushroom spines and a decrease in stubby spines to the level observed in healthy mice. Several of these improvements were also observed with overexpressing Bcl-2^K17D^ except that the difference between WT and 5xFAD mice in parameters of mushroom and stubby spines percentages remains statistically significant.

### Analysis of the number of amyloid plaques in the brain of 5xFAD mice

**Fig. 6:**
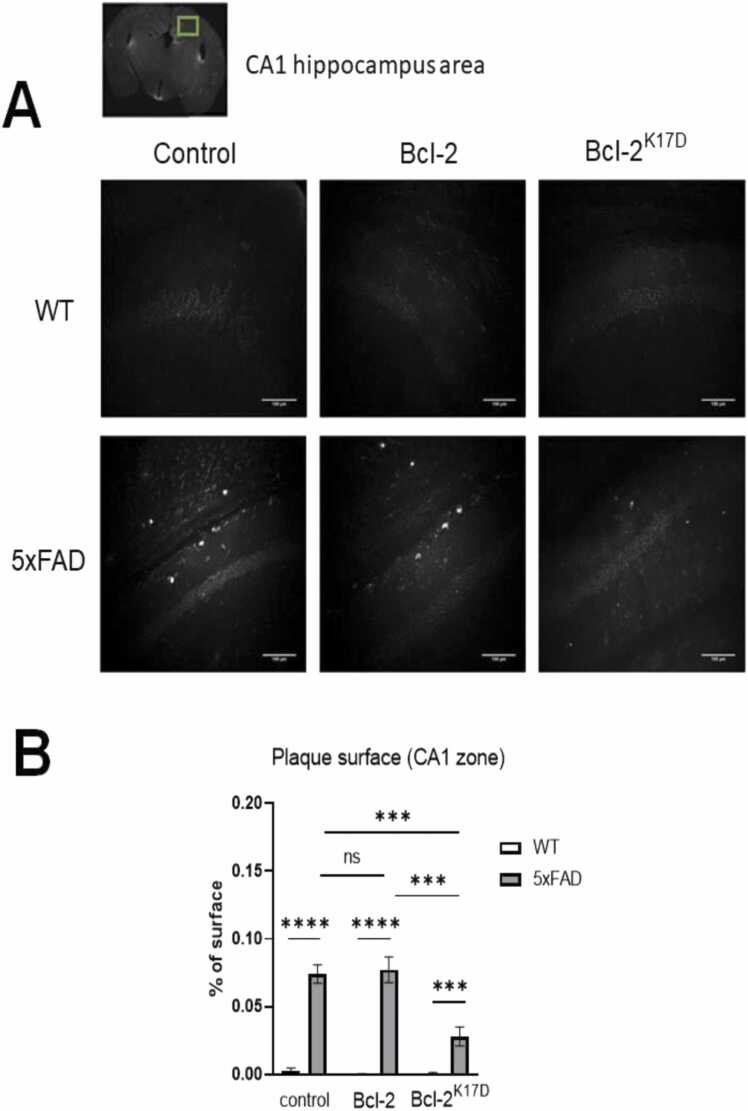
Bcl-2^K17D^ decreases the amount of amyloid plaques in the CA1 zone of the hippocampus**.** The result of counting the number of amyloid plaques in the CA1 region of the hippocampus of WT and 5xFAD mice injected with Control, 3xFLAG-Bcl-2-P2A-mCherry, 3xFLAG-Bcl-2^K17D^-P2A-mCherry viral vector (in each group n = 18). A) Representative microphotos of immunohistochemical staining with 6E10 antibodies of 100 µm brain sections in the CA1 observation zone in all experimental groups. The scale - 100 µm. C) Histogram of the percentage area occupied by plaques in CA1 area in all experimental groups. Results are presented as mean±SEM. ns – no statistical detection, **** p < 0.0001,*** p < 0.0005.

**Fig. 7:**
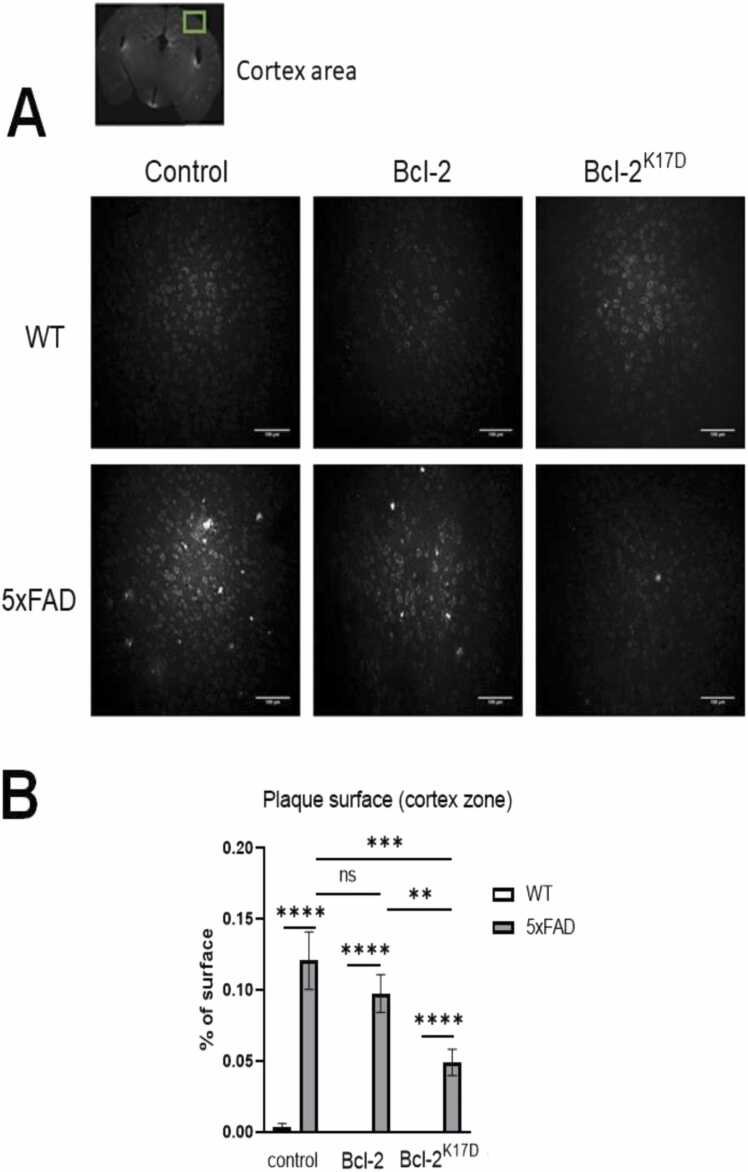
Bcl-2^K17D^ decreases the amount of amyloid plaques in the cortex zone. The result of counting the number of amyloid plaques in the cortex region of WT and 5xFAD mice injected with Control, 3xFLAG-Bcl-2-P2A-mCherry, 3xFLAG-Bcl-2^K17D^-P2A-mCherry viral vector (in each group n = 18). A) Representative microphotos of immunohistochemical staining with 6E10 antibodies of 100 µm brain sections in the cortex observation zone in all experimental groups. The scale - 100 µm. C) Histogram of the percentage area occupied by plaques in cortex area in all experimental groups. Results are presented as mean±SEM. ns – no statistical detection, * ** * p < 0.0001,*** p < 0.0005, ** p < 0005.

Amyloid plaque counts were made on 100 µm brain slices made from 6 months old 5xFAD and WT mice injected with control vector, Bcl-2 and Bcl-2^K17D^ at 3.5 months. Three zones were selected for calculation - CA1 and CA3 hippocampus areas and the area of the cortex located laterally above CA1. Based on the results of this experiment, it became clear that the count of amyloid plaques in the hippocampus CA3 zone does not reflect the obvious features of the 5xFAD transgenic line, which is consistent with our previous studies (Chernyuk et al., 2016), therefore, it was decided to count only the hippocampus CA1 and cortex areas. The results are presented in Fig. 6, Fig. 7 for each of the selected zones. In 5xFAD mice injected with the control vector in the hippocampus CA1 and in the cortex areas, the percentage surface occupied by amyloid plaques is 0.074 ± 0.007 % (n = 18) and 0.121 ± 0.020 % (n = 18), respectively. In 5xFAD mice with Bcl-2 overexpression, these values were 0.077 ± 0.010 % (n = 18) and 0.097 ± 0.013 % (n = 18), respectively. After injection of the viral vector encoding the Bcl-2^K17D^ mutant, a significant reduction in the number of amyloid plaques occurred in both zones under study, to 0.028 ± 0.07% in the CA1 area and to 0.049 ± 0.009% in the cortex area. From the data obtained, it can be concluded that the overexpression of the native Bcl-2 protein does not have amyloid-protective properties, in contrast to its mutant form Bcl-2^K17D^, which significantly suppresses amyloid plaque formation in the cortex and the hippocampus.

## Discussion

The main finding of this work is that elevating the Bcl-2-protein levels induces synaptoprotective properties in AD by suppressing excessive Ca^2+^ signals and thus is a promising strategy to counteract the development of AD features. Overexpression of wild-type Bcl-2 in the hippocampus CA1 area of 5xFAD mice reduces the loss of mushroom spines and restores their number to the level of healthy mice. The Bcl-2^K17D^ mutant, which has impaired binding to the IP_3_R, also displayed synaptoprotective properties, but its effect is less pronounced than that of the native form. Protein interaction analysis revealed that Bcl-2 binds to both IP_3_R1 and RyR2. Instead, Bcl-2^K17D^ mutant is less effective in interacting with IP_3_R1 compared to wild-type Bcl-2, while this mutant interacts with RyR2 with similar efficiencies as wild-type Bcl-2. This is consistent with our previous studies (Rong et al., 2009, Monaco et al., 2012, Vervliet et al., 2014), demonstrating that Bcl-2^K17D^ is impaired in IP_3_R binding but not in RyR binding. However, our previous work focused on the modulation of RyR1 and RyR3 channels by Bcl-2 proteins, while this work now was extended towards RyR2. Moreover, this work also for the first time addresses the relevance of Lys17 in Bcl-2 for binding IP_3_Rs versus RyRs in primary hippocampal neurons. Overall, our results reveal that using AAV-based expression approaches in hippocampal neurons that Bcl-2, expressed in hippocampal neurons using AAV-based approaches, can interact with endogenous, neuronal IP_3_R1 and RyR2 channels. Furthermore, mutating Lys17 into Asp also impairs the binding of Bcl-2 to IP_3_R1, while it does not affect Bcl-2′s ability to bind to RyR2. Since this is a novel molecular finding, we validated this in HEK293 T-Rex cells ectopically expressing RyR2 demonstrating that both wild-type Bcl-2 and Bcl-2^K17D^ are equally potent in inhibiting RyR2 activity, analyzed as caffeine-evoked [Ca^2+^] rises.

The Bcl-2 protein is found in the ER, mitochondria, or nuclear membrane - organelles that play a key role in the intracellular Ca^2+^ homeostasis (Yang et al., 2018). We found that in hippocampal neurons Bcl-2 forms clusters in the soma. An analysis of the literature showed that such an arrangement of Bcl-2 is characteristic of its form associated with the ER (Ciechomska et al., 2009) or the outer mitochondrial membrane (Jiao et al., 2005). Phosphorylated form of Bcl-2-Ser70, which is observed in various cells (Zhou et al., 2017, Chong et al., 2020), also forms clusters. Phosphorylation of Bcl-2 is necessary for the realization of its antiapoptotic function (Ruvolo et al., 2001). There is also evidence that deletion of the BH4 domain transforms Bcl-2 into a proapoptotic protein (Rong et al., 2009). Our result showed that Bcl-2 and IP_3_R1, which are both present in the form of clusters, is characterized by low level of co-localization, but by using expansion microscopy we found that some are lying close to each other, and therefore are able to form contacts. Based on the obtained data, we may assume that these proteins are located in different but contacting organelles – Bcl-2 is localized in the form clusters in mitochondria at the MAMs (Yang et al., 2018) where it binds to IP_3_R1, which is localized in the ER of the neuron. Further studies are required to unravel the nature and function of Bcl-2 protein clusters.

In this study, the effect of Bcl-2 and Bcl-2^K17D^ overexpression on the amount of amyloid plaques in the 5xFAD mice brains was tested. It turned out that only the Bcl-2^K17D^ has amyloid-protective properties suggesting that selective inhibition of RyRs while sparing IP_3_Rs by Bcl-2 is important in this process. Several research groups have pointed to a role for RyR in the dysregulation of Ca^2+^ signaling, as neuronal expression of RyR has been increased in various AD mouse models and cell lines (Chan et al., 2000, Stutzmann et al., 2006, Bruno et al., 2011, Chakroborty et al., 2012, Bussiere et al., 2017). Treatment with dantrolene, a RyR inhibitor, has been reported to normalize impaired ER Ca^2+^ signaling and reduce Aβ deposits (Chakroborty et al., 2012). These data are also consistent with our results, and we can suggest that, in a 5xFAD mouse model of Alzheimer's disease, inhibition of RyR2-dependent Ca^2+^ exit from the ER alone leads to a greater therapeutic effect than blocking both RyR2 and IP_3_R1 channels.

## Conclusions

Overall, our work indicates that Bcl-2-based strategies offer potential to prevent adverse AD-related outcomes, though their beneficial impacts are rather complex. Both Bcl-2 and Bcl-2^K17D^ mutant possess synaptoprotective properties, though at the level of neuronal morphology, wild-type Bcl-2 provided more potent protection. Yet, when analyzing their effect on amyloid plaques, the main pathophysiological feature of AD, the Bcl-2^K17D^ mutant provided a superior response to amyloid plaque accumulation compared to wild-type Bcl-2. At the level of synaptic contacts, the blocking of two Ca^2+^ release channels from the ER is sufficient to reduce defects in AD, but at the tissue level, the positive synaptoprotective effect may be leveled by other functions of the native form of Bcl-2. Thus, we hypothesize that the interaction of Bcl-2^K17D^ with RyR2 channels thereby suppressing excessive RyR2 activity while sparing IP_3_R1 channel has beneficial properties in a 5xFAD mouse model, representing the familial form of AD. However, to prove the importance of targeting RyR2 through Bcl-2 will require the identification of the key molecular determinants in Bcl-2 critical for binding and inhibiting RyR channels. Developing a Bcl-2 mutant that fails to bind to RyRs will be key to determine whether the protective effects of Bcl-2 and/or Bcl-2^K17D^ are mediated through the inhibition of RyRs. Nevertheless, our results indicate that the potential of Bcl-2-based strategies against AD and warrant follow-up research to elucidate the importance of the RyR2/Bcl-2 axis in AD.

## Funding

This work was supported by the 10.13039/100000002National Institutes of Health grant R56AG071310 (IB), by the 10.13039/501100006769Russian Science Foundation grant No. 22-15-00049 (IB), by the Bijzonder Onderzoeks Fonds KU Leuven (C14/19/099) (GB), the Research Foundation—Flanders (10.13039/501100003130FWO; project numbers G0E7520N (GB) and G081821N (GB) and fellowship numbers: 11K7122N (MC) and 12ZG121N (TV)); Stichting Alzheimer Onderzoek (SAO IP_3_ receptor) (GB). We are grateful to Wayne S.R. Chen (University of Calgary, Canada) for kindly providing the HEK293 T-Rex RyR2 cells.

## CRediT authorship contribution statement

The idea of the work and the theoretical basis of the study (I. Bezprozvanny, T. Vervliet, G. Bultynck), planning of experiments (I. Bezprozvanny, G. Bultynck, D. Chernyuk, T. Vervliet, E. Pchitskaya and M. Callens), design, generation & validation of adeno-viral vectors (Leuven Viral Vector Core, C. Van den Haute, G. Bultynck, M. Callens, T. Vervliet), implementation experiments on co-immunoprecipitation (D. Chernyuk), experiments of Ca^2+^ signaling in HEK cells (M. Callens), experiments on co-localization of proteins (E. Pchitskaya and M. Chigriai), 5xFAD-M hybrid line breeding (A. Ilina), experiments on the analysis of the morphology of dendritic spines (D. Chernyuk and A. Gordeev), performing experiments on the analysis of the number of amyloid plaques (D. Chernyuk and M. Polozova), data processing (D. Chernyuk, E. Pchitskaya and M. Callens), writing and editing the manuscript (I. Bezprozvanny, G. Bultynck, D. Chernyuk, E. Pchitskaya, T. Vervliet and M. Callens).

## Declarations of interest

IB is a member of scientific advisory board of Neurodon, LLC and receives compensation. GB and IB are part of the FWO Scientific Research Network CaSign (W001422N).
